# The frequency and correlates of complex post-traumatic stress disorder among patients being treated for borderline personality disorder: cross-sectional study

**DOI:** 10.1192/bjo.2025.10838

**Published:** 2025-11-12

**Authors:** Kirsten Barnicot, Mike Crawford

**Affiliations:** Department of Global, Public and Population Health and Policy, City St George’s University of London, UK; Department of Psychiatry, Imperial College London, UK

**Keywords:** Personality disorders, trauma and stressor-related disorders, mental health services, general adult psychiatry, diagnosis and classification

## Abstract

**Background:**

Despite overlapping diagnostic criteria and aetiology, the frequency of complex post-traumatic stress disorder (C-PTSD) in people being treated for borderline personality disorder (BPD) is unknown.

**Aims:**

To establish the frequency and correlates of probable C-PTSD in people meeting the diagnostic criteria and being treated for BPD.

**Method:**

C-PTSD was assessed in 87 patients meeting the diagnostic criteria for BPD and initiating treatment in out-patient personality disorder services in the UK, using the Structured Clinical Interview for DSM-IV Axis II Personality Disorders diagnostic interview, items from the Structured Interview for Disorders of Extreme Stress – Self Report and other measures. The cross-sectional association between C-PTSD and demographics, trauma and clinical variables was evaluated with logistic, ordinal and linear regression.

**Results:**

A total of 93% of participants reported a trauma history (95% CI 88–98%), and 57% met the criteria for probable C-PTSD (95% CI 47–67%). Previous sexual trauma increased the odds of probable C-PTSD (odds ratio 6.22, 95% CI 2.21–17.54, *P* < 0.001). Probable C-PTSD was associated with an increased odds of self-harm in the past 12 months (odds ratio 9.41, 95% CI 1.87–47.27, *P* = 0.01) and higher levels of abandonment fears (odds ratio 2.78, 95% CI 1.17–6.55, *P* = 0.02), abandonment–avoidant behaviour (odds ratio 4.25, 95% CI 1.30–13.91, *P* = 0.02) and identity instability (odds ratio 4.39, 95% CI 1.79–10.78, *P* < 0.01).

**Conclusions:**

C-PTSD symptoms are likely to be common in people diagnosed with BPD, and are associated with higher overall psychiatric severity, with potential implications for formulation and treatment.

Experiences of childhood neglect and trauma, and post-traumatic stress disorder (PTSD), are very common among people diagnosed with borderline personality disorder (BPD) who receive treatment in mental health services.^1–3^ For instance, a study in the USA found that 61% of in-patients meeting the diagnostic criteria for BPD reported childhood sexual abuse, and 59% reported childhood physical abuse.^1^ Studies of out-patients diagnosed with BPD who were receiving dialectical behaviour therapy in the USA and UK have found that 56 and 57%, respectively, met the diagnostic criteria for PTSD.^2,3^ In the USA study, both women who did and did not meet the diagnostic criteria for PTSD had a high lifetime incidence of physical and sexual violence, with on average five lifetime incidents of physical assault in the PTSD group and three in the non-PTSD group, and on average 16 lifetime incidents of sexual assault in the PTSD group and nine in the non-PTSD group.^3^ The first incident occurred on average at age 7 years in the PTSD group and age 9 years in the non-PTSD group. Rates of trauma and PTSD are lower in people who meet the diagnostic criteria for BPD as identified through screening and interview assessment of general population samples (e.g. PTSD 32.0%),^4,5^ potentially because individuals in these studies represent a less severely affected population who do not necessarily seek help or merit referral to mental health services.

Although trauma is an important risk factor for developing all types of mental health difficulties, BPD is three times more strongly associated with a history of trauma than other psychiatric diagnoses, including mood disorders, other personality disorders and psychosis.^5^ It has been argued that BPD should be reconceptualised as a complex trauma response, driven in part by the view that attributing trauma survival responses to personality is stigmatising and iatrogenic.^6,7^ Other authors point to a multifactorial aetiological model, where experiences of trauma are an important – but not sole – risk factor for developing the difficulties associated with BPD, in dynamic interaction with genetic, epigenetic and other psychosocial risk factors.^8^

Complex post-traumatic stress disorder (C-PTSD) is a new diagnosis in the ICD-11, characterised by both classic PTSD symptoms (having experienced an event or series of events of an extremely threatening or horrific nature; re-experiencing the traumatic event or events in the form of vivid intrusive memories, flashbacks or nightmares; avoidance of internal or external reminders of the traumatic events; persistent perceptions of heightened current threat) and ‘disturbances in self-organisation’ (DSO) (see Table 1).^9^ C-PTSD DSO and BPD share near-identical difficulties with emotional dysregulation (Table 1).^9–11^ Although both diagnoses are also characterised by difficulties with interpersonal relationships and self-concept, a key difference has been argued to lie in the stability of these difficulties. C-PTSD is thought to be characterised by stable avoidance of emotional closeness in contrast with the volatility in relationships associated with BPD; and by a stably negative self-concept in contrast to the unstable self-concept associated with BPD.^12,13^ Although trauma as an aetiological factor is a diagnostic criterion for C-PTSD, this is not the case for BPD.^9–11^ Yet multifactorial aetiological models are also important to consider in the case of C-PTSD, since it is known that temperamental and psychosocial risk factors modify the risk of developing PTSD symptoms following trauma.^14,15^

**Table 1:** Comparison of the diagnostic criteria for complex post-traumatic stress disorder disturbances of self-organisation and borderline personality disorder/personality disorder with borderline pattern

Domain	ICD-11^9^ diagnostic criteria for C-PTSD disturbances of self-organisation^a^	DSM-IV/DSM-5^10,11^ diagnostic criteria for BPD and ICD-11^9^ diagnostic criteria for PD-BP^b^^,^^c^
Emotional dysregulation	Severe and persistent problems in affect regulation. Examples include Heightened emotional reactivity to minor stressors Violent outbursts Reckless or self-destructive behaviour Dissociative symptoms when under stress Emotional numbing, particularly the inability to experience pleasure or positive emotions	Emotional instability owing to marked reactivity of mood Inappropriate intense anger or difficulty controlling anger manifested in frequent displays of temper A tendency to act rashly in states of high negative affect, leading to potentially self-damaging behaviours Recurrent episodes of self-harm Transient dissociative symptoms or psychotic-like features (e.g. brief hallucinations, paranoia) in situations of high affective arousal Chronic feelings of emptiness
Self-concept	Beliefs about oneself as diminished, defeated or worthless, accompanied by feelings of shame, guilt or failure related to the traumatic event	Identity disturbance, manifested in markedly and persistently unstable self-image or sense of self
Relationships with others	Difficulties in sustaining relationships and in feeling close to others	Frantic efforts to avoid real or imagined abandonment A pattern of unstable and intense interpersonal relationships, which may be characterised by vacillations between idealisation and devaluation, typically associated with both strong desire for and fear of closeness and intimacy

C-PTSD, complex post-traumatic stress disorder; BPD, borderline personality disorder; PD-BP, personality disorder, borderline pattern.

a. A person meeting the diagnostic criteria must manifest at least one example of criteria 1, 2 and 3.

b. A person meeting the diagnostic criteria must manifest at least five of criteria 1 to 9.

c. The diagnostic criteria for the DSM-IV/DSM-5 BPD and ICD-11 PD-BP are identical and the listed criteria encapsulate all three diagnostic manuals.

Two latent class analyses have found that individuals seeking treatment for trauma who endorsed high levels of BPD traits, were also moderately likely to endorse classic PTSD symptoms, and were as likely as those in the C-PTSD class to endorse C-PTSD DSO.^13,16^ This highlights the potential overlap between the diagnoses. However, individuals seeking treatment for trauma may differ from the population of patients who are referred for personality disorder treatment by mental health services. It is important to determine the frequency of C-PTSD in people being treated for BPD by mental health services.

We aimed to establish the frequency of probable ICD-11 C-PTSD in patients being treated for BPD by mental health services and confirmed to meet the diagnostic criteria for BPD by a researcher-administered diagnostic interview, and to determine the cross-sectional association with trauma type; self-harm; BPD traits relating to fear of abandonment, unstable interpersonal relationships and unstable identity; and overall BPD severity.

## Method

### Participants

Clinicians in participating services invited all patients initiating treatment at six out-patient personality disorder services in London and Southampton, UK, between March 2014 and September 2016, to participate in a wider study evaluating outcomes of dialectical behaviour therapy and mentalisation-based therapy.^17^ The included services all offered dialectical behaviour therapy or mentalisation-based therapy to patients deemed to have a personality disorder. We included patients who met the diagnostic criteria for BPD according to a Structured Clinical Interview for DSM-IV Axis II Personality Disorders (SCID-II) diagnostic interview conducted by the first author,^18^ and who had a clinical diagnosis of BPD (coded as ICD-10 emotionally unstable personality disorder). Patients judged by the clinical team to have intellectual disability or difficulty communicating in English of sufficient severity to prevent completion of study questionnaires, and/or insufficient capacity to provide informed consent, were ineligible to participate.

### Procedure

The authors assert that all procedures contributing to this work comply with the ethical standards of the relevant national and institutional committees on human experimentation and with the Helsinki Declaration of 1975, as revised in 2013. The UK National Health Service (NHS) Research Ethics Service Committee South East Coast – Surrey approved all procedures involving human patients (reference number 14_LO_0158). The first author obtained written informed consent from all patients. Subsequently, the first author conducted a SCID-II interview,^18^ to ascertain that participants met the diagnostic criteria for BPD, followed by the measures outlined below.

### Operationalisation of C-PTSD

We operationalised probable C-PTSD as meeting interviewer-rated diagnostic criteria for ICD-11 PTSD, and meeting self-reported diagnostic criteria for ICD-11 C-PTSD DSO (emotion dysregulation, negative self-concept and difficulties in relationships) on a proxy measure. The first author assessed ICD-11 PTSD by using the self-reported Traumatic Antecedents Questionnaire to assess trauma history,^19^ and a subset of items from the PTSD module of the Structured Clinical Interview for DSM-IV Axis I Disorders.^20^ We used this approach because ICD-11 diagnostic criteria for PTSD are identical to the DSM-IV criteria other than exclusion of numbing of general responsiveness and some indicators of hyperarousal (difficulties sleeping, irritability, difficulty concentrating). As the current gold standard self-report measure for assessment of ICD-11 C-PTSD DSO, the DSO subscale of the International Trauma Questionnaire (ITQ-DSO),^21^ had not been developed at the time of this study, we assessed C-PTSD DSO by constructing a proxy measure using near-identical items from the following self-report measures: The Structured Interview for Disorders of Extreme Stress (SIDES-SR) (developed to assess an earlier conceptualisation of C-PTSD),^22^ the Post-Traumatic Stress Disorder Symptom Scale (PSS)^23^ and the State Shame and Guilt Scale (Table 2).^24^

**Table 2:** Assessment of complex post-traumatic stress disorder disturbances in self-organisation

ICD-11 C-PTSD DSO criteria	ITQ-DSO Items	Proxy ITQ-DSO questionnaire items used in the current study
Severe and pervasive problems in affect regulation. Examples include heightened emotional reactivity to minor stressors, violent outbursts, reckless or self-destructive behaviour, dissociative symptoms when under stress, and emotional numbing, particularly the inability to experience pleasure or positive emotions	Score ≥2 on ≥1 item 1. When I am upset it takes me a long time to calm down 2. I feel numb or emotionally shut down	≥1 of the following: 1. SIDES-SR item 3. When I feel upset, I have trouble finding ways to calm myself down (score ≥2) 2. PSS item 11. Feeling emotionally numb (unable to cry or have loving feelings) (score ≥2)
Persistent beliefs about oneself as diminished, defeated or worthless, accompanied by deep and pervasive feelings of shame, guilt or failure related to the stressor. For example, the individual may feel guilty about not having escaped from or succumbing to the adverse circumstance, or not having been able to prevent the suffering of others	Score ≥2 on ≥1 item 3. I feel like a failure 4. I feel worthless	≥1 of the following: 3. SIDES-SR item 26. I feel that I have something wrong with me after what happened, that can never be fixed (score ≥2) 4. SGGS item 5. I’ve felt worthless and powerless. (score ≥3)
Persistent difficulties in sustaining relationships and in feeling close to others. The person may consistently avoid, deride or have little interest in relationships and social engagement more generally. Alternatively, there may be occasional intense relationships, but the person has difficulty sustaining them	Score ≥2 on ≥1 item 5. I feel distant or cut-off from people 6. I find it hard to stay emotionally close to people	≥1 of the following: 5. SIDES-SR item 29. I feel set apart and very different from most people (score ≥2) 6. SIDES-SR item 32. I avoid having relationships with other people (score ≥2)

C-PTSD, complex post-traumatic stress disorder; DSO, disturbances in self-organisation; ITQ-DSO International Trauma Questionnaire Disturbances in Self-Organisation; SIDES-SR, Structured Interview for Disorders of Extreme Stress – Self Report; PSS, Post-traumatic Stress Disorder Symptom Scale; SSGS, State Shame and Guilt Scale.

### Other measures

The first author assessed gender identity and ethnicity with a standardised demographic questionnaire developed for the study, and enumerated incidents of self-harm in the previous 12 months by using the Suicide Attempt Self-Injury Interview (SASII).^25^ Self-harm was operationalised as ‘Any overt, acute, nonfatal self-injurious act where both act and bodily harm or death are clearly intended (i.e. both the behavioural act and the injurious outcomes are not accidental) that results in actual tissue damage, illness, or, if no intervention from others, risk of death or serious injury’.^26^ Participants reported the extent to which they were affected by different BPD traits using the Borderline Evaluation of Severity Over Time (BEST) questionnaire.^27^ Individual BPD traits are rated on a five-point scale from 1 (not at all) to 5 (extremely), and summed to provide a total score ranging from 12 to 72.

### Analysis

We calculated the proportion of the sample meeting the diagnostic criteria for ICD-11 PTSD, probable ICD-11 C-PTSD DSO and probable ICD-11 C-PTSD, with 95% confidence intervals. We also calculated the proportion of people meeting the diagnostic criteria for PTSD but not C-PTSD DSO, and *vice versa*, with 95% confidence intervals. We used logistic, ordinal logistic or linear regression as appropriate, in Stata/SE (version 14.2 for Windows; Timberlake, London, UK; https://timberlake.co/uk/), to compare demographic, trauma and clinical variables between participants who met the criteria for C-PTSD and those who did not.

## Results

**Fig. 1:**
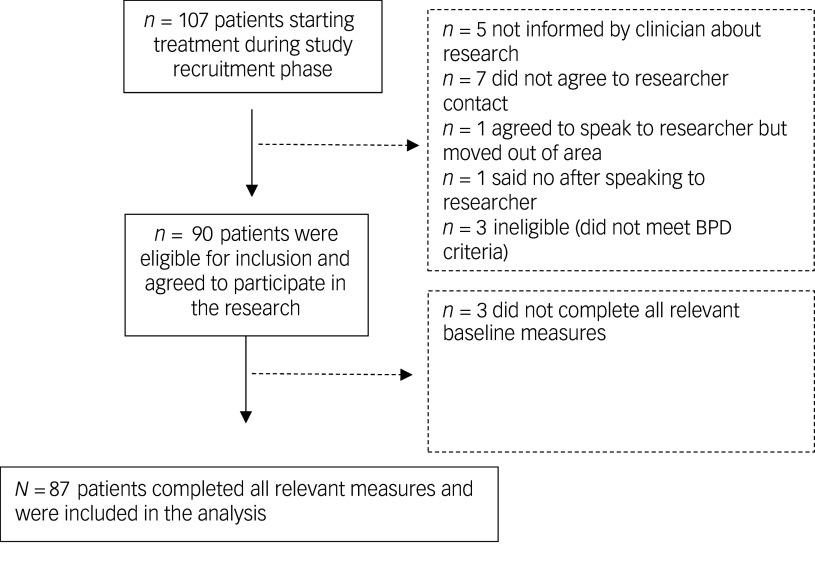
Flow of patients through the study. BPD, borderline personality disorder.

Participant flow through the study is shown in Fig. 1. Consent rates for the wider study were high (92% of eligible patients approached), and 87 out of 90 participants provided data for the present analysis. The sample consisted of 63 people identifying as female and 24 identifying as male, aged between 18 and 71 years; 64% were White and 36% were Black, Asian or minority ethnic.

A total of 93% of the sample (95% CI 88–98%) reported having experienced one or more traumatic events as defined in the ICD-11 diagnostic criteria for PTSD, including sexual trauma (63%), non-sexual violence (68%), witnessing violence or the sudden death of a close person (54%), narrowly escaping death (1%) or being threatened with violence (1%). Where information on trauma timing and frequency was available in medical notes, most commonly participants had experienced at least one of the above types of trauma during childhood (88% of 59 cases with data), primarily before adolescence (64% of 59 cases with data), and most commonly the trauma had been a repeated event (73% of 66 cases with data). Sixty-eight per cent of participants met the diagnostic criteria for PTSD (95% CI 58–78%), 78% of participants met the criteria for C-PTSD DSO 95% CI (69–87%) and 57% of participants met tbe diagnostic criteria for both PTSD and probable C-PTSD DSO, indicating probable C-PTSD (95% CI 47–67%). A further 21% of participants met the criteria for probable C-PTSD DSO, but not for PTSD (95% CI 12.5–29.5%), whereas 10% met the diagnostic criteria for PTSD, but not for C-PTSD DSO (95% CI 4–16%). Of those with a trauma history, a history of sexual trauma significantly increased the odds of meeting the criteria for probable C-PTSD (odds ratio 6.22, 95% CI 2.21–17.54, *P* < 0.001; Table 3). Meeting the criteria for probable C-PTSD significantly increased the odds of scoring more highly on the BEST self-report items ‘Worrying that someone important in your life is tired of you or planning to leave you’ (odds ratio 2.78, 95% CI 1.17–6.55, *P* = 0.02), ‘Going to extremes to try to keep someone from leaving you’ (odds ratio 4.25, 95% CI 1.30–13.91, *P* = 0.02) and ‘Extreme changes in how you see yourself. Shifting from feeling confident about who you are to feeling like you are evil, or that you don’t even exist’ (odds ratio 4.39, 95% CI 1.79–10.78, *P* < 0.01), compared with participants who did not meet the criteria. Patients with probable C-PTSD were also significantly more likely to have self-harmed in the past 12 months (odds ratio 9.41, 95% CI 1.87–47.27, *P* = 0.01). There was no evidence of a difference between participants who did and did not meet the criteria for C-PTSD in the incidence of other types of trauma, childhood trauma and repeated trauma, or in self-reported unstable relationships and overall BPD severity.

**Table 3:** Association between probable complex post-traumatic stress disorder and demographic and clinical characteristics

Variables	Met the criteria for probable C-PTSD *n* = 50	Reported a trauma history^a^ and did not meet the criteria for probable C-PTSD *n* = 31	Odds ratio or B-statistic (95% CI)	*P*-value
Gender identity, *n* (%)				
Male	16 (32)	8 (26)	0.79 (0.29–2.16)	0.64
Female	34 (68)	23 (74)		
Sexual trauma, *n* (%)	41 (82)	14 (45)	6.22 (2.21–17.54)	<0.001
Non-sexual violence, *n* (%)	36 (72)	23 (74)	0.96 (0.35–2.68)	0.94
Witnessing violence, *n* (%)	27 (54)	20 (65)	0.68 (0.27–1.70)	0.41
Childhood trauma, *n* (%)	32 (65)^b^	20 (100)^c^	Non-estimable^d^	Non-estimable^d^
Repeated trauma, *n* (%)	33 (79)^e^	15 (65)^f^	1.96 (0.63–6.06)	0.25
Self harm in past 12 months, *n* (%)	46 (92)	22 (71)	9.41 (1.87–47.27)	0.01
BPD severity (BEST score), mean (s.d.)	45.2 (8.73)	41.6 (11.35)	1.04 (0.99–1.09)	0.12
BEST: Worrying that someone important in your life is tired of you or planning to leave you, mean (s.d.)	3.30 (1.36)	2.84 (1.27)	2.78 (1.17–6.55)	0.02
BEST: Going to extremes to try to keep someone from leaving you, mean (s.d.)	1.84 (1.32)	4.25 (1.25)	4.25 (1.30–13.91)	0.02
BEST: Major shifts in your opinions about others such as switching from believing someone is a loyal friend or partner to believing the person is untrustworthy or unhelpful, mean (s.d.)	3.20 (1.34)	3.42 (1.29)	1.18 (0.51–2.72)	0.70
BEST: Extreme changes in how you see yourself. Shifting from feeling confident about who you are to feeling like you are evil, or that you don’t even exist, mean (s.d.)	3.92 (1.26)	2.97 (1.40)	4.39 (1.79–10.78)	0.001

C-PTSD, complex post-traumatic stress disorder; BPD, borderline personality disorder; BEST, Borderline Evaluation of Severity Over Time.

a. History of experiencing sexual trauma, non-sexual violence and/or witnessing violence.

b. *n* = 39 with data on trauma timing.

c. *n* = 20 with data on trauma timing.

d. Non-estimable because trauma occurred in childhood for all participants with a trauma history, no C-PTSD and available data.

e. *n* = 42 with data on trauma frequency.

f. *n* = 23 with data on trauma frequency.

## Discussion

This is, to our knowledge, one of the first assessments of the frequency of probable C-PTSD among individuals meeting the diagnostic criteria for BPD who also have a clinical diagnosis of BPD and are being treated for personality disorder by mental health services. Over half of patients (57%) met the diagnostic criteria for probable C-PTSD. Participants meeting the criteria for both BPD and C-PTSD were more likely to report experiencing sexual trauma and to have a recent history of self-harm, and reported more abandonment fears, abandonment–avoidant behaviour, and identity instability, compared with participants with BPD who did not meet the criteria for C-PTSD. Participants meeting the criteria for both diagnoses did not differ significantly in self-reported unstable perceptions of interpersonal relationships, although this variable was non-significantly higher in this group.

### Interpretation of findings and comparison with existing literature

This was a highly trauma-exposed sample, with 93% of patients reporting having experienced traumatic event(s) as defined in the ICD-11 diagnostic criteria for PTSD, most commonly sexual trauma or violence. As in previous studies of trauma-exposed individuals, C-PTSD was more prevalent than classic PTSD alone.^28^ The 57% rate of C-PTSD we have identified among people with a clinical BPD diagnosis falls in-between the C-PTSD rates of 45% and 68% identified in previous UK and USA studies, which included patients who did not necessarily have a clinical diagnosis of BPD, but were assessed by an interviewer to meet the diagnostic criteria.^13,29^

We found that sexual trauma was particularly strongly associated with C-PTSD, and with the much smaller subset of participants experiencing PTSD without DSO, compared with those without PTSD symptoms. This is consistent with findings from prior studies in which sexual violence was linked to a greater likelihood and severity of PTSD symptoms compared with non-sexual violence.^30–32^ We found no evidence of an association between C-PTSD and age at trauma onset, consistent with prior findings that severe interpersonal trauma in childhood or adulthood is equally likely to result in C-PTSD.^33^ We also found no evidence of an association between C-PTSD and repeated trauma. One explanation for this lies in the fact that the majority of our participants with a BPD diagnosis had a history of repeated sexual and/or violent trauma, most commonly originating in childhood, and thus this factor did not differentiate well between BPD with and without C-PTSD. However, these analyses were limited by missing data on trauma onset and frequency.

Although C-PTSD and BPD share near-identical diagnostic criteria for emotional dysregulation, they have been theorised to be differentiated by stasis versus lability in interpersonal relationship difficulties and in self-concept.^12,13^ Yet, in our data, indices of interpersonal instability including abandonment fears and abandonment–avoidant behaviour, and identity instability, were significantly higher among participants meeting the criteria for both BPD and C-PTSD compared with participants who met the criteria for BPD and not C-PTSD. The identified odds ratios suggest that this could be a clinically significant effect. One interpretation of this finding is that the distinction between C-PTSD as characterised by stable negative interpersonal and identity functioning, and BPD as characterised by interpersonal and identity instability, breaks down when a person meets the diagnostic criteria for both. Certainly, both C-PTSD and BPD are characterised by dissociation, which is also strongly linked to sexual abuse and thought to contribute, at least in part, to the identity difficulties seen in people diagnosed with BPD.^9,34^ Alternatively, perhaps the measures used do not adequately capture the stasis versus lability aspects of these constructs. Additionally, in alignment with their increased likelihood of engaging in self-harming behaviour, perhaps endorsing diagnostic criteria for both BPD and C-PTSD is a hallmark of worse overall psychiatric symptom severity. The wide confidence intervals suggest uncertainty in ascertaining the true magnitude of this difference. The uncertainty may stem from the wide standard deviations on the relevant BPD interpersonal instability item scores, which in turn, relates to the widely recognised heterogeneity within the BPD diagnosis whereby there are 256 different ways in which one can meet five of the nine possible diagnostic criteria.^35^

### Implications for clinical practice and further research

The findings imply that C-PTSD symptoms should routinely be considered in people diagnosed with personality disorder with borderline pattern or BPD and treated by mental health services. On the basis of our findings, C-PTSD symptoms may be linked to higher risk behaviours such as self-harm, emphasising the importance of assessing and treating them. The clinical utility of assigning both diagnoses should be critically considered. ICD-11 recommends that, where an individual meeting the diagnostic criteria for C-PTSD also meets diagnostic criteria for a personality disorder, the latter should only be assigned where clinically useful.^8^

Two-thirds of the patients in our sample who did not meet the diagnostic criteria for PTSD did report C-PTSD DSO symptoms, i.e. emotional dysregulation, avoidance and alienation in relationships and a negative self-concept. Thus, it seems that although virtually all of our sample had a history of trauma, and the majority had responded to this in ways that met the diagnostic criteria for C-PTSD, a substantial minority had responded in ways that are theorised to reflect trauma-related responses, but did not meet all of the C-PTSD criteria. This potentially calls into question the utility of these diagnostic constructs, which place strict definitions around classification of someone’s experiences as either a trauma-related disorder or a personality-related disorder. In clinical practice, it is important to look beyond the binary parameters of diagnosis and to formulate each individual’s particular strengths and difficulties, with an exploration of the personal relevance of trauma, adversity and other aetiological factors in shaping their development through the life course.^36^

Current evidence-based psychological interventions for BPD, such as dialectical behaviour therapy and mentalisation-based therapy, are present-focused and do not directly address trauma or PTSD symptoms.^37,38^ By contrast, multicomponent trauma-focused and exposure-based interventions to directly address PTSD symptoms, are recommended for PTSD and C-PTSD.^39–41^ Randomised controlled trials in the USA and Germany have found that trauma-focused interventions can safely and effectively be combined with dialectical behaviour therapy for dual-diagnosed individuals, achieving high PTSD remission rates.^42–44^ The effectiveness of psychological interventions in addressing C-PTSD DSO is currently unclear. Although interventions such as dialectical behaviour therapy and mentalisation-based therapy are known to help with the behavioural consequences of emotional dysregulation, such as self-harm and substance misuse, little research has evaluated their effectiveness for ameliorating internal experiences of emotional instability.^39,45^ Cognitive–behaviour therapy, exposure-based therapies and eye movement desensitisation reprocessing therapy have been found effective for improving C-PTSD-aligned interpersonal difficulties and negative self-concept.^39^ It is unclear whether the identity and interpersonal instability associated with BPD requires a different treatment approach, nor how treatment effectiveness differs for patients experiencing both sets of difficulties. In the UK, trauma survivors given a diagnosis of BPD are campaigning for trauma-focused treatment pathways that are entirely separated from the personality disorder construct.^7^ It is important to take into account the diagnostic and treatment preferences of each individual. Beyond specific interventions, trauma-informed care is an approach that is thought to be helpful for trauma survivors transdiagnostically, and is widely used in the UK and USA.^41^ It is a set of principles that can run alongside both present-focused and trauma-focused interventions. Key aspects include understanding, where relevant, how people’s experiences and behaviours have been shaped by coping with trauma and adversity, and using this understanding to formulate a compassionate approach to their difficulties.^46,47^ Other key aspects are enabling access to trauma-focused interventions where this is needed and wanted, and creating an environment that prevents re-traumatisation by avoiding coercion, increasing trustworthiness and transparency, offering collaboration and empowerment, and creating a sense of safety.^46,47^

Further research on the overlap between BPD and C-PTSD should draw on both clinical and non-clinical samples, where the extent of overlap may differ. Additionally, the ICD-11 has only recently been implemented in the World Health Organization member states. In contrast to the DSM-IV/DSM-5 criteria for BPD used in the present study, the ICD-11 has further blurred the distinction between BPD and C-PTSD by stating in the diagnostic guidelines for personality disorder with borderline pattern that ‘some individuals with a borderline pattern may also believe themselves to be inadequate, bad, guilty, disgusting, and contemptible’ (akin to the stable negative self-concept associated with C-PTSD), and that ‘some individuals with a borderline pattern may feel profoundly different and isolated from other people, may feel a painful sense of alienation and pervasive loneliness…..and may have problems in establishing and maintaining consistent and appropriate levels of trust in interpersonal relationships’ (akin to C-PTSD stable difficulties in sustaining relationships and feeling close to others).^9^ It will be important to determine how the availability of the new ICD-11 C-PTSD diagnosis affects patients with a BPD diagnosis; in particular, how many people are given an additional C-PTSD diagnosis or are re-diagnosed altogether. Further research is required to better understand the impact of classifying a person’s difficulties as BPD versus C-PTSD. Assignation of the latter diagnosis may arguably be associated with less stigmatising responses by health professionals and with better access to trauma-focused interventions, where these are wanted and deemed clinically appropriate. By contrast, concerns have been raised that trauma survivors with a BPD diagnosis who do not fit within the narrow parameters of the C-PTSD diagnostic criteria may encounter a two-tier system, where access to trauma-based formulations of their experiences and/or trauma-focused interventions is desired but denied.^36^ Finally, it will be helpful to evaluate whether taking a trauma-informed approach alongside offering evidence-based interventions, where relevant, can improve patient satisfaction and treatment outcomes.

### Strengths and limitations

The findings are strengthened by the high consent rate and representative sample of patients referred to multiple UK community personality disorder services. The use of validated semi-structured diagnostic interviews to ascertain BPD and PTSD diagnoses is a further strength. The main weakness was the use of an analogue self-report measure to assess C-PTSD DSO symptoms. Additionally, information on trauma timing and frequency was not available for the full sample.

In conclusion, a high proportion of patients with a BPD diagnosis in mental health services are likely to also meet the diagnostic criteria for C-PTSD. A further substantial minority are likely to have experienced trauma and to meet some, but not all, diagnostic criteria for C-PTSD. The findings imply that C-PTSD symptoms should be routinely considered in people diagnosed with BPD. The implications of co-occurring C-PTSD symptoms for case formulation and treatment should be carefully considered, in line with the recommendations in the National Institute for Health and Care Excellence guidelines that patients with PTSD should be offered evidence-based interventions that directly address PTSD symptoms. Beyond arbitrary diagnostic bifurcations, it is possible that a transdiagnostic trauma-informed approach to working with trauma survivors, alongside evidence-based interventions, may be helpful.

## Data Availability

The data that support the findings of this study are available from the corresponding author, K.B., upon reasonable request.
